# Highly Dispersed Blast Furnace Sludge as a Source of Iron and Zinc for Sugar Beet: Effects on Oxidative Stress Markers and Micronutrient Bioaccumulation

**DOI:** 10.3390/ijms27125243

**Published:** 2026-06-10

**Authors:** Olga V. Zakharova, Natalia S. Strekalova, Inna A. Vasyukova, Dmitrii S. Suvorov, Bekzod B. Khaydarov, Igor N. Burmistrov, Alexander A. Gusev

**Affiliations:** 1 Scientific and Educational Center for Environmental Science and Biotechnology, Derzhavin Tambov State University, 392000 Tambov, Russia; olgazakharova1@mail.ru (O.V.Z.); kotova-ns@yandex.ru (N.S.S.); vasyukovaia@gmail.com (I.A.V.); suvorov.ds@misis.ru (D.S.S.); bekzod1991@mail.ru (B.B.K.); glas100@yandex.ru (I.N.B.); 2 Department of Functional Nanosystems and High-Temperature Materials, National University of Science and Technology (MISIS), 119991 Moscow, Russia

**Keywords:** blast furnace sludge, micro-dispersed particles, iron and zinc, sugar beet, oxidative stress markers (PPO; POD), metal bioaccumulation

## Abstract

Blast furnace sludge is a micro- and nano-dispersed metallurgical waste rich in iron and zinc, yet its accumulation poses a serious environmental challenge. Here we demonstrate its potential as a source of iron and zinc for sugar beet (*Beta vulgaris* L.), a crop with high micronutrient demand and economic importance. At application rates of 0.5–2 t ha^−1^ in alluvial-meadow soils with neutral pH, the sludge increased root yield by up to 1.5-fold and sugar content by up to 1.4-fold compared to untreated controls. The optimal dose (0.1 g kg^−1^ in greenhouse) significantly reduced the activity of oxidative stress markers—polyphenol oxidase (PPO) by 7.5-fold and peroxidase (POD) by 8-fold—indicating alleviation of cellular stress. The sludge also exhibited phytoprotective properties, reducing leaf necrosis under field conditions. A single application at these rates posed no food safety risks: lead and cadmium levels in beetroots and soil remained below international regulatory limits, and zinc accumulation in beetroots (≤10 mg kg^−1^) was an order of magnitude below the FAO/WHO guideline. However, repeated annual applications would gradually increase soil zinc; preliminary screening suggests that applying 2 t ha^−1^ annually could approach the soil MPC within 4–5 years under a linear accumulation scenario, necessitating long-term monitoring.

## 1. Introduction

The metallurgical industry is one of the most significant sources of industrial waste [1]. The main types of waste generated during the smelting of iron and steel include dust, sludge, slag, scale, etc. [2]. Since this waste can pose a serious threat to ecosystems, its safe disposal is of vital importance [1]. At the same time, metal-containing waste is highly valuable for reuse. For example, highly dispersed blast furnace sludge, which is produced during wet cleaning of blast furnace gases, is characterized by a high iron content, as well as the presence of metals such as zinc, magnesium, manganese, copper, etc. [3,4]. However, these wastes, which are potentially rich in iron metallurgical raw materials, cannot be used in the sintering process without additional processing due to their high zinc content (more than 0.5%), which destroys the lining of blast furnaces [5]. Therefore, many metallurgical enterprises dispose of waste by placing it in sludge storage facilities and dumps.

According to data from the World Steel Association, global steel production in 2024 amounted to 1882.6 million tons, while blast furnace iron production amounted to 1.265 billion tons [6,7]. Considering that the production of each ton of cast iron usually generates about 6 kg of sludge [8,9], more than 7 million tons of this waste are generated annually worldwide. From the perspective of a closed-loop economy, the disposal of sludge in storage facilities is not only harmful to the environment but also economically unfeasible: annual global losses of iron amount to about 2 million tons, carbon to about 2 million tons, and the losses of zinc amount to 280,000 tons [9]. In view of the above, the development of technologies for the use of blast furnace sludge as a source of iron and zinc, in particular, is highly relevant.

For example, according to Custom Market Insights, growing demand for high-quality agricultural products, combined with active efforts by industry players to introduce innovative products, will contribute to the expansion of the fertilizer market. In particular, the market for iron preparations for plant cultivation is expected to reach USD 645.55 million by 2032 [10]. As for zinc, the global market for zinc sulfate for plant cultivation is expected to reach USD 2.95 billion by 2033 [11].

Iron is an essential element for normal plant growth and development, as it participates in biochemical reactions and processes as a non-protein component of a number of enzymes [12]. In addition, iron influences the formation of the structure and functioning of chloroplasts, as well as the synthesis of chlorophyll [13]. Zinc is also an important trace element for plants, as it is involved in many metabolic and physiological processes in the cell [14,15]. Furthermore, zinc promotes dry matter accumulation [16] and increases the sugar content of sugar beets [17].

As already mentioned, blast furnace sludge has a rich microelement composition, but its frequent content of toxic metals (Pb, Cd, Hg, As, etc.) [18] poses certain risks when used in crop production Iron in blast furnace sludge is typically present as oxides (Fe_2_O_3_, Fe_3_O_4_, FeO) and zinc ferrite (ZnFe_2_O_4_); Zn, in addition to ferrite, can also be present as oxide (ZnO) and sulfide (ZnS). Other components include SiO_2_, CaO, MgO, etc. [19,20]. The blast furnace sludge samples are neutral to slightly alkaline (pH 7.6–9) [21], allowing their use without the risk of soil acidification.

There exist only a few studies [22,23,24,25] that explore the possibility of using blast furnace sludge in agriculture. At the same time, for example, metallurgical slags containing calcium compounds have long been successfully used as soil improvers that can effectively deacidify soils [26,27,28,29].

A critical feature of the studied sludge is its high dispersion with a significant submicron fraction [30]. Such fine particles possess a large specific surface area, which can accelerate the dissolution of metal-bearing phases in the soil solution compared to coarse materials [31,32,33]. This, in turn, is expected to enhance the short-term bioavailability of iron and zinc for plant roots, making the sludge not merely a pollutant but a reactive micronutrient carrier.

Therefore, in this study, we combined agronomic, biochemical, and food safety assessments to investigate the effect of highly dispersed blast furnace sludge on sugar beet. Specifically, we aimed to: (1) determine the optimal application rate for yield and sugar content; (2) evaluate the sludge’s impact on key oxidative stress markers, PPO and POD, as indicators of plant physiological status; (3) quantify the accumulation of iron, zinc, and toxic metals (Pb, Cd) in plant organs and soil; (4) assess the long-term safety of repeated applications. This integrated approach allows us to link the beneficial effects to potential molecular mechanisms involving micronutrient-driven stress mitigation. To address these questions, we conducted greenhouse dose–response experiments followed by field trials at three application rates. We measured growth parameters, photosynthetic productivity, PPO and POD activities, as well as metal accumulation in soil and beet tissues.

## 2. Results

### 2.1. Results of Blast Furnace Sludge Research

**Figure 1:**
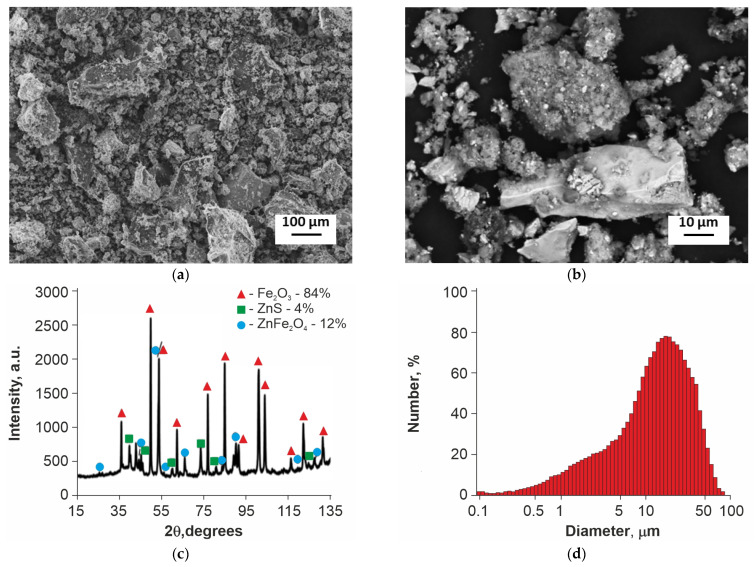
Characterization of the sludge sample: (**a**) and (**b**) electron micrographs; (**c**) diffractogram; (**d**) particle size distribution.

SEM analysis showed that the studied metallurgical sludge sample is a finely dispersed powder consisting of both agglomerates and individual particles with irregular morphology and varying dispersibility (Figure 1a,b). The size of the sludge particles and aggregates ranges from 100 nm to 200 μm.

Analysis of the phase composition of the sample showed that iron in the sample is mainly present in the form of oxide (Fe_2_O_3_) and zinc ferrite (ZnFe_2_O_4_) (Figure 1c). Zinc is also present in the form of sulfide. It was found that the size of the sludge particles ranges from 0.1 to 100 μm (Figure 1d), which allows the sludge to be classified as micro- and nanodispersed waste. The size distribution of the particles is close to bimodal, with a median distribution d50 of approximately 14 μm. Notably, a significant fraction of particles is in the submicron range, which may have implications for their reactivity and bioavailability.

The elemental analysis of the sludge is presented in Table 1. As can be seen from the results, the sludge sample is characterized by a high content of iron and zinc, 41.56 and 8.4 wt%, respectively. The sludge also contains elements that are important for plant growth and development, such as potassium, phosphorus, and sulfur.

Thus, the examined blast furnace sludge sample is a highly dispersed material with particle sizes ranging from 100 nm to 100 μm. The main elements of the sludge are iron, zinc, oxygen, carbon, and silicon. Small amounts (<5%) of sodium, calcium, aluminum, and sulfur are present. Less than 1% of phosphorus, potassium, and titanium have been detected.

### 2.2. Greenhouse Experiment Results

**Figure 2:**
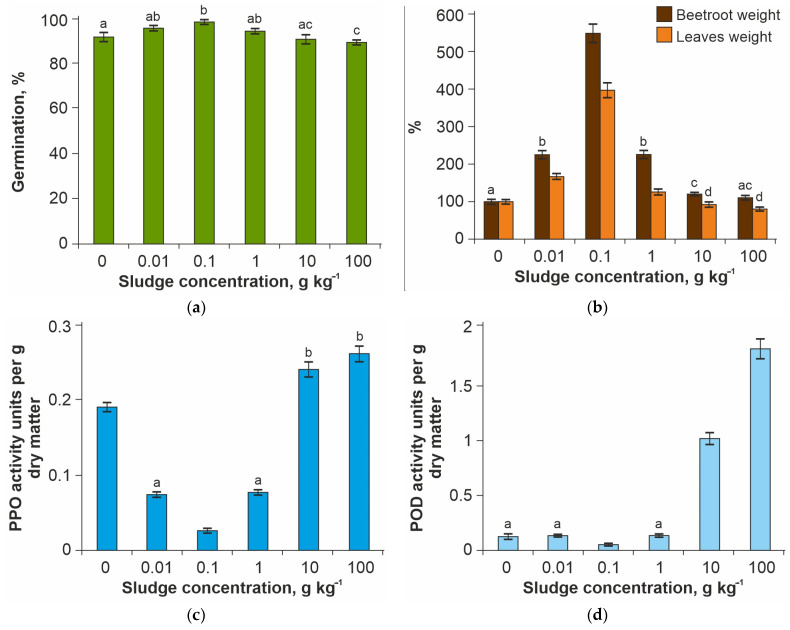
Effect of blast furnace sludge on beet plants in the greenhouse experiment: (**a**) germination; (**b**) plant fresh weight, *n* = 60 in triplicate; (**c**) PPO activity (*n* = 9 in triplicate); (**d**) POD activity (*n* = 9 in triplicate). Values followed by the same letter are not significantly different.

Analysis of the results of the greenhouse experiment revealed a distinct dose-dependent response of sugar beet plants to sludge concentration, identifying a clear toxicity threshold above 10 g kg^−1^ (Figure 2). At the low dose of 0.1 g kg^−1^ concentration, beet germination increased by 5% compared to the control (Figure 2a). The other sludge doses below 100 g kg^−1^ did not have any significant effect on the indicator. In the case of the maximum concentration, a 3% decrease in germination was observed. The maximum plant weight was also observed in the 0.1 g kg^−1^ variant, with the average root weight being 5.5 times greater than that of the control plants and the leaf weight being 4 times greater (Figure 2b). Sludge doses of 0.01 and 1 g kg^−1^ also had a significant effect on plant development. In these cases, the average increase in root mass was 123%, and the leaf mass increased by 63% and 23%, respectively. However, exceeding the 1 g kg^−1^ threshold had a negative effect on root development, with root mass decreasing by 10% at 10 g kg^−1^ of waste in the substrate and by 20% at 100 g kg^−1^, signaling the onset of phytotoxicity.

As can be seen from Figure 2c, the activity of the PPO enzyme, which oxidizes phenolic compounds and participates in adaptive responses to negative environmental factors, demonstrated a biphasic dose-dependent pattern. It was lowest in the leaves of plants cultivated at 0.1 g kg^−1^ of sludge, where it was 7.5 times lower than in the extract from the control plants, suggesting an alleviation of baseline nutritional stress. At the same time, increasing the dose beyond the optimal range to 10 and 100 g kg^−1^ increased enzyme activity by 1.3 and 1.4 times compared to the control, respectively. At 0.01 and 1 g kg^−1^ of sludge in the substrate, PPO activity was 2.5 times lower than in the control.

POD activity in beet leaves followed a similar dose-dependent trend. It was also minimal at 0.1 g kg^−1^ of sludge, whereas at 10 and 100 g kg^−1^, enzyme activity reached maximum values exceeding the control values by 8 and 14 times and confirming a severe stress response (Figure 2d).

Thus, during the greenhouse experiment, a clear dose-dependent effect was established: while low doses up to 1 g kg^−1^ provided significant growth stimulation and minimized oxidative stress indicators, exceeding this concentration marked a toxicity threshold, resulting in a pronounced stress response and the suppression of root development.

### 2.3. Field Experiment Results

As mentioned earlier, application rates of 0.5, 2, and 4 t ha^−1^ were selected for the field trial. The dose of 0.5 t ha^−1^ was calculated based on an average soil density of ~1.3–1.4 g cm^−3^ and corresponds to a concentration of 0.1 g kg^−1^ in the greenhouse experiment, which showed the greatest positive effect.

**Figure 3:**
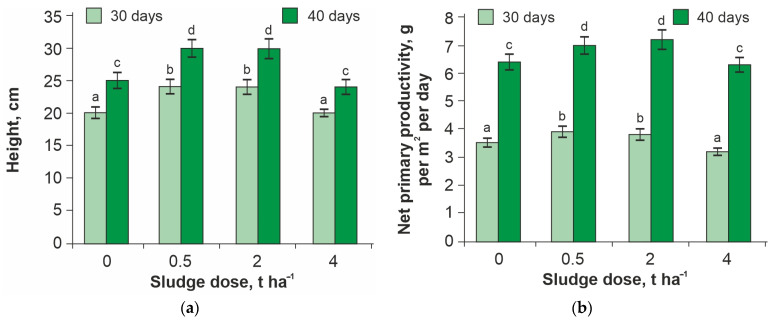
Results of interim accounting of indicators: (**a**) height of plants (*n* = 50 in triplicate); (**b**) net photosynthetic productivity (*n* = 3 in triplicate). Values followed by the same letter are not significantly different.

One month after planting, an interim assessment of growth and photosynthetic productivity indicators was carried out (Figure 3). The second interim assessment was carried out 40 days after planting.

As can be seen from the diagrams (Figure 3a), the metallurgical sludge analyzed had a beneficial effect on the morphophysiological indicators of plants, demonstrating a distinctly non-linear response pattern. In the experimental groups of 0.5 and 2 t ha^−1^, an increase in plant height and net photosynthetic productivity was observed. Conversely, further doubling the application rate to 4 t ha^−1^ did not have a significant effect on the recorded indicators, marking the upper limit of the useful application range for these traits.

**Figure 4:**
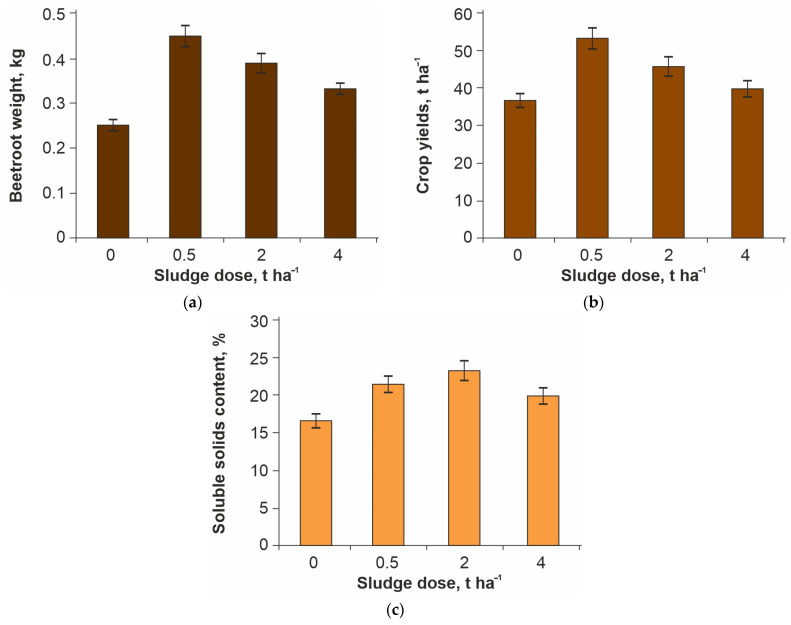
Indicators of biological and economic productivity of crops (after 130 days of cultivation): (**a**) beetroots weight (*n* = 50 in triplicate); (**b**) crop yields (*n* = 5 in triplicate); (**c**) soluble solids content in beetroots (*n* = 9 in triplicate).

After 130 days of cultivation, the final indicators of biological and economic productivity of the crop were recorded. A significant increase in the mass of beetroots was found when the soil was treated with 0.5 and 2 t ha^−1^ of sludge (Figure 4a). With the minimum dose of sludge, the indicator increased by 1.8 times, and with 2 t ha^−1^ the root crop weight exceeded the control by 1.6 times. It is worth noting that the maximum dose of waste of 4 t ha^−1^ also had a stimulating effect on weight gain.

The maximum yield was also observed at 0.5 t ha^−1^ of sludge (Figure 4b). Thus, in this variant, the yield was 53.2 t ha^−1^, which is almost 1.5 times higher than in the variant without sludge treatment. At 2 t ha^−1^, the yield was also higher than the control, on average by 1.2 times. Further increasing the waste application rate to 4 t ha^−1^ led to a decline in efficiency, having no effect on the analyzed indicator compared to the control. This non-linear performance underscores that higher doses do not yield proportional agronomic benefits. Unlike other indicators, the maximum sugar content of root crops, amounting to 23.1%, which is 1.4 times higher than in the control, was observed in plants cultivated with 2 t ha^−1^ of sludge in the soil (Figure 4c). In the 0.5 t ha^−1^ variant, the indicator exceeded the control by 1.3 times, and in the 4 t ha^−1^ variant, by 1.2 times. In general, it can be noted that blast furnace sludge has a favorable effect on sugar accumulation in beet roots, albeit with decreasing efficiency at the highest dose.

ANOVA revealed significant effects of sludge application on plant height, net photosynthetic productivity, root weight, yield, and sugar content (*p* < 0.05). Tukey’s HSD test confirmed that the doses of 0.5 and 2 t ha^−1^ were significantly superior to the control and to the 4 t ha^−1^ dose for most parameters (Figure 3 and Figure 4), statistically validating the non-linear pattern of the crop response and helping define the optimal safe utilization thresholds.

**Figure 5:**
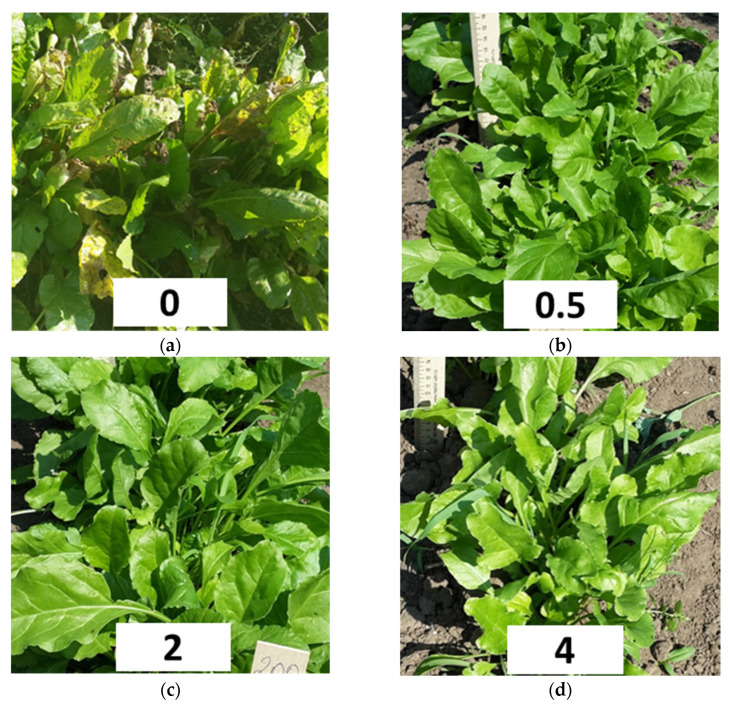
Appearance of plants (day 90 of the experiment): (**a**) control; (**b**) 0.5 t ha^−1^; (**c**) 2 t ha^−1^; (**d**) 4 t ha^−1^ of sludge.

During the observation of the growth, a large number of necrotic spots were noted on the leaves of the control plants, while no necrosis was observed on the plants treated with sludge (Figure 5). Although this phenomenon has been described only qualitatively rather than quantitatively, it may nevertheless indicate that the sludge has phyto-protective properties, the mechanisms of which require further study.

In addition, as can be seen in Figure 5, the beet leaves in the control sample were partially yellow in color, possibly due to Fe deficiency. The high iron content in the sludge probably compensated for the iron deficiency.

At the end of the experiment, the heavy metal content in the plant samples and soil from the experimental plots was analyzed. Among the most hazardous components of the sludge, zinc was analyzed. Iron content in plant organs was also assessed (Table 2). Furthermore, despite their absence in the waste, lead and cadmium levels were measured in plants, as well as manganese, copper, cobalt, and chromium (III) in the soil. It is known that some metals can affect the levels of others, both in plants and soil [34].

The study of plant samples did not reveal any effect of blast furnace sludge application on the lead and cadmium content in plant organs (Table 2). At the same time, regardless of the sludge application rate, zinc accumulation in beet leaves and root crops was noted. An increase in the iron content in beet leaves was also noted, with the maximum (more than 2 times more than in the control variant) noted with the minimum dose of sludge.

Analysis of the content of regulated heavy metals in the soil from experimental plots showed an increase in zinc concentration proportional to the increase in sludge dose (Table 3). It should be noted that even in the variant with the maximum dose of sludge, no exceedance of maximum permissible concentration (MPC) standards was recorded. For the other elements, no significant effect of sludge on their content in the soil was detected.

Field research has shown that blast furnace sludge has potential as a source of micronutrients for sugar beet cultivation, operating within a well-defined, non-linear efficacy window. Adding sludge at the optimal range of 0.5–2 t ha^−1^ can not only increase crop yields, but also boost sugar output and act as a plant protection agent, whereas higher rates (4 t ha^−1^) yield diminishing returns. Importantly, even at the highest tested rate, the concentrations of lead and cadmium in the harvested beetroots (Table 2) remained below the Maximum Permissible Levels established for food products, confirming the safety of the crop for consumption under the tested application rates. Thus, while the safe application range extends up to 4 t ha^−1^ from an ecotoxicological perspective, the useful agronomic range is restricted to 0.5–2 t ha^−1^ due to the non-linear response layout. However, given the observed increase in zinc content in plant organs, the safe use of sludge in crop production necessitates systematic monitoring of zinc levels in plants and long-term monitoring of its accumulation in the soil.

## 3. Discussion

The results of this comprehensive study demonstrate that highly dispersed blast furnace sludge, traditionally considered a problematic metallurgical waste, can be effectively repurposed as a micronutrient source for sugar beet cultivation, thereby offering a sustainable recycling pathway. Unlike organic amendments such as compost or biochar, which primarily improve soil structure and provide macronutrients [38,39], the sludge is a purely mineral product. Its agronomic value lies almost exclusively in its content of microelements, particularly Fe, Zn, and Si, making it a low-cost alternative to conventional synthetic micronutrient fertilizers (e.g., chelates or sulfates). By valorizing a large-tonnage waste stream, this approach embodies the principles of circular economy and green metallurgy. From a regulatory perspective, under the conditions of this study, the analyzed sludge meets the contaminant limits for lead and cadmium established by international standards such as the EU Fertilising Products Regulation (EU 2019/1009) [40] and the FAO/WHO guidelines [41], supporting its potential use as a safe fertilizer component in a single-application scenario.

The observed stimulation of growth, biological and economic productivity of sugar beet at optimal sludge doses (0.5–2 t ha^−1^ in the field) is strongly associated with the high iron and zinc content of the material. Iron is a key micronutrient in photosynthesis: in plant shoot tissues, 80–90% of iron is contained in chloroplasts, where it functions in iron-sulfur clusters and heme cofactors [42]. Furthermore, iron is an essential component of cytochromes in the mitochondrial electron transport chain, where it supports root respiration and energy-dependent processes, directly contributing to root growth and biomass accumulation. Iron deficiency directly impairs chlorophyll synthesis and the assembly of pigment-protein complexes, leading to chlorosis and reduced photosynthetic capacity [43,44]. Our field data support this link: control plants exhibited yellowing leaves and significantly lower leaf iron concentrations (18.2 mg kg^−1^) compared to sludge-treated plants (up to 52.6 mg kg^−1^ at 0.5 t ha^−1^), demonstrating that sludge application effectively corrected iron deficiency. Zinc plays an equally important role in carbohydrate metabolism and sugar accumulation. Through its involvement in the biosynthesis of tryptophan, a precursor of the auxin indoleacetic acid, zinc promotes root elongation and overall plant growth [45,46], which aligns with the increased root biomass observed in our study. Furthermore, zinc participates in energy production and the regulation of sugar storage in roots [17], providing a plausible explanation for the significant increase in sugar content (up to 1.4 times) recorded in sludge-treated beets. While direct comparisons with conventional Fe/Zn fertilizers or soil availability fractions were not analyzed, the heavy enrichment of these elements and their mineral forms in the sludge suggests they are key drivers of the growth response. The species-specific response observed when comparing sugar beet with our previous study on rapeseed [23], where the optimal dose was 2 t ha^−1^ for rapeseed but 0.5 t ha^−1^ for sugar beet yield, further underscores that physiological demands for micronutrients vary between crops.

In addition to its direct nutritional function, the sludge demonstrated pronounced phytoprotective properties, acting as a multi-component stress protector. While the role of Fe and Zn in growth has already been discussed, their involvement in the antioxidant system, as well as the presence of silicon (as silicates), may play a role here. Field observations revealed markedly fewer necrotic spots on leaves of sludge-treated plants compared to controls, suggesting enhanced stress tolerance. This protective effect was particularly evident under the arid conditions that characterized the field trial period. Greenhouse analyses provided indirect support for this observation: at the optimal sludge dose (0.1 g kg^−1^), the activity of oxidative stress indicators, PPO and POD, was significantly reduced (7.5-fold and 8-fold, respectively) compared to control plants, suggesting a mitigation of oxidative stress [47,48]. Although direct markers of oxidative damage, several components of the sludge likely contribute to this observed response. First, zinc is known to stabilize cell membranes and act as a cofactor for antioxidant enzymes such as superoxide dismutase [47,49]. Second, the sludge contains over 6% silicon, which enhances resistance to environmental stresses, including drought, nutrient deficiency, and disease [50,51,52,53,54]. Silicon increases cell wall rigidity through biosilicification [52,53] and has been specifically shown to alleviate water deficit stress in sugar beet [54]. Moreover, recent evidence suggests that Zn and Fe can function not only as enzyme cofactors but also as signaling molecules that prime plant defense responses, potentially activating systemic acquired resistance pathways [55,56]. Thus, blast furnace sludge may act as a multi-component stress ameliorant, complementing its nutritional value.

A key factor that may contribute to the agronomic effectiveness of blast furnace sludge at relatively low application rates (0.5–2 t ha^−1^) is its high degree of dispersity. As shown in Figure 1d, the sludge particle size distribution ranges from 100 nm to 100 μm, with a significant fraction in the submicron range and a median diameter (d50) of approximately 14 μm. Such fine particles possess a high specific surface area, which is hypothesized to accelerate the weathering and dissolution of metal-bearing phases (e.g., Fe_2_O_3_, ZnFe_2_O_4_) in the soil solution compared to their coarse-grained counterparts [57,58]. This increased surface reactivity likely enhances the short-term bioavailability of essential micronutrients like Fe and Zn to plant roots, but this proposed mechanism, however, requires future experimental validation. Soil properties such as pH, organic matter content, and texture can significantly influence the bioavailability of sludge-derived micronutrients and the mobility of potential contaminants like zinc [59]. In acidic soils, for instance, the solubility of zinc increases, which could elevate plant uptake and the risk of phytotoxicity, whereas in calcareous soils, iron availability may be further limited, potentially altering the fertilizer’s efficacy [60]. Therefore, extrapolation of our results to other edaphic and climatic contexts should be undertaken with caution. Another aspect not addressed in field trials is the potential interaction of sludge components with the soil microbiome. Iron and zinc are essential cofactors for many microbial enzymes involved in organic matter decomposition, nutrient cycling, and the synthesis of plant growth-promoting substances [13,61]. While our study did not include microbiological analyses, previous research suggests that micronutrient amendments can stimulate the abundance and activity of rhizosphere bacteria and fungi, thereby improving nitrogen availability and root health [62]. This potential pathway warrants further investigation. Additionally, the oxidation-reduction activity of iron can affect the availability of other elements such as phosphorus, which often forms insoluble precipitates with iron oxides [63]. Although the sludge iron is predominantly in the ferric (Fe^3+^) state, root exudates and microbial activity can create localized reducing conditions, potentially leading to partial reduction to more soluble ferrous (Fe^2+^) forms, the primary form for plant uptake [64]. In addition to microbial pathways, the rhizosphere chemical environment directly drives the crop’s physiological response, especially regarding iron bioavailability under alkaline or calcareous soil conditions where native iron is largely immobilized. As a dicotyledonous species, sugar beet actively overcomes these constraints via the classical Strategy I mechanism, which relies on root-induced proton extrusion to locally lower pH and ferric chelate reductase activity [65]. The exceptional specific surface area of the nano- and micro-dispersed sludge particles facilitates this uptake by continuously releasing labile iron phases in close proximity to the root zone. Once absorbed, the metabolic role of this iron extends from chloroplasts to the mitochondrial electron transport chain and the activation of iron-dependent superoxide dismutases (Fe-SODs), which are crucial for maintaining cellular redox homeostasis [66]. Beyond serving as a source of micronutrients, the sludge particles themselves may act as physical habitats for microbial colonization, potentially accelerating their dissolution and the release of Fe and Zn through biofilm-mediated processes. Collectively, these considerations highlight that the agronomic effectiveness of blast furnace sludge emerges from complex interactions between the material’s characteristics and the receiving environment, reinforcing the need for multi-location trials.

A critical aspect of the work was assessing the food safety of products grown using sludge. Analysis of heavy metal accumulation demonstrated that a single sludge application at the recommended rates did not compromise crop quality or soil integrity. The concentrations of toxic elements, including lead and cadmium, in both harvested beetroots and soil remained at control levels and substantially below the maximum permissible limits established by international sanitary norms and regulations, TP TC 021/2011, Regulation (EU) 2023/915, and SanPiN 1.2.3685-21 [35,36,37]. Furthermore, zinc concentrations in the beetroots did not exceed 10 mg kg^−1^, remaining an order of magnitude below the FAO/WHO food safety guideline (100 mg kg^−1^) [41], thereby confirming consumer safety. The principal long-term consideration for sustained use is the accumulation of zinc in the arable layer. While the observed dose-dependent increase in soil zinc content (from 3.6 mg kg^−1^ in control to 10.6 mg kg^−1^ at 4 t ha^−1^) remained well below the established maximum permissible concentration of 23 mg kg^−1^ following a single application, repeated annual applications could lead to gradual accumulation over time. As a preliminary screening-level estimate based on the linear accumulation pattern observed in our one-year study (approximately +4.6 mg kg^−1^ per 2 t ha^−1^ application), annual application at 2 t ha^−1^ could approach the MPC within four to five years. However, it is important to note that this simplified projection does not account for critical mitigating factors such as crop removal, leaching, shifting soil pH, organic matter dynamics, or changes in metal speciation over time, which may slow down or accelerate this process. This highlights the need for systematic long-term monitoring, identifying zinc as the critical element requiring long-term observation and establishing the need for a precautionary management framework rather than constituting a fundamental limitation to the technology’s viability. It should also be noted that zinc tends to accumulate in surface soil layers due to its relatively low mobility in neutral to alkaline conditions, which is relevant for crop rotation planning and tillage practices.

It is worth noting that, in addition to directly introducing micronutrients, sludge can also affect soil composition indirectly. Despite the absence of copper and cobalt in the original sludge, their accumulation was recorded at the maximum dose (4 t ha^−1^). This phenomenon may be explained by competitive sorption processes: metals can compete for adsorption sites in soil, and the presence of high concentrations of iron and zinc can displace other cations, potentially increasing their mobility or, conversely, their retention [34,67]. More specifically, iron oxides, which constitute the main component of blast furnace sludge, are known to act as effective sorbents for heavy metals, accumulating copper and cobalt through adsorption and incorporation into the mineral structure [68,69,70]. Soils rich in iron oxides and hydroxides (hematite, goethite) may contain increased amounts of cobalt, since Co^2+^ is capable of replacing Fe^3+^ or Fe^2+^ in the crystal lattice of these minerals [71]. The sorption properties of blast furnace sludge are confirmed by the work of López-Delgado et al., which demonstrated efficient sorption of Pb^2+^, Zn^2+^, Cd^2+^, Cu^2+^, and Cr^3+^ on sludge [72]. This observation reinforces the value of comprehensive post-harvest soil analysis, as it reveals indirect geochemical effects that would not be predicted from sludge characterization alone. On the other hand, some components of the sludge can also have a positive physical and chemical effect on the soil. In particular, calcium and magnesium oxides impart a mild liming effect to the material [28,73]. Consequently, unlike some nitrogen-based fertilizers, the application of this sludge is unlikely to contribute to soil acidification and may even offer a mild ameliorative effect against acidity. This is consistent with the slightly alkaline pH (7.4–8.2) of the native sludge supernatant, suggesting that the material itself possesses an inherent liming potential, further supporting its suitability for use in acidic agricultural soils where both micronutrient deficiency and low pH often co-occur.

In summary, this study demonstrates that micro- and nano-dispersed blast furnace sludge holds strong potential as a multi-component amendment, providing bioavailable iron and zinc while appearing to mitigate cellular oxidative stress, as suggested by decreased PPO and POD activities. The research demonstrates that optimal application rates of 0.5–2 t ha^−1^ significantly enhance key agronomic parameters, increasing root crop yield by up to 1.5 times and sugar content by up to 1.4 times compared to untreated controls. These are strongly linked to the synergistic action of iron, zinc, and silicon, whose specific nutritional and potential protective functions have been detailed above. Beyond its nutritional function, the sludge exhibits notable phytoprotective properties, as evidenced by reduced leaf necrosis in the field and decreased activity of oxidative stress markers in greenhouse experiments. The micro- and nanoscale dispersion of the sludge is hypothesized to influence its functional benefit, potentially facilitating the gradual release of iron and zinc, increasing their phytoavailability without the rapid development of toxicity. While further direct measurements of ROS and lipid peroxidation are required to confirm the precise physiological pathways, the observed reduction in PPO and POD activities points toward an alleviation of cellular oxidative stress, providing a plausible link to the improved crop yields. Critically, under the conditions of this study, a single application at the recommended rates does not compromise crop quality or soil integrity, with lead and cadmium concentrations remaining at control levels and zinc concentrations in beetroots staying an order of magnitude below international food safety guidelines. The principal long-term consideration concerns zinc accumulation in soil, which requires monitoring if sludge is applied annually. Future research should include long-term field trials across diverse soil types and crops, standardized characterization protocols for sludge batches, and assessment of effects on soil microbiota and nutrient bioavailability. Given the annual global generation of blast furnace sludge exceeding 7 million tons, elucidating the underlying molecular and physiological pathways—including the regulation of oxidative stress markers and metal transporters—is a necessary step for its safe and large-scale agricultural utilization.

## 4. Materials and Methods

### 4.1. Characterization of Blast Furnace Sludge

**Figure 6:**
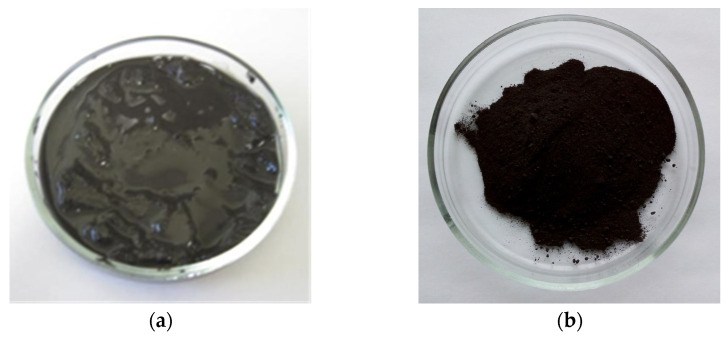
Sample of blast furnace sludge: (**a**) after sampling; (**b**) after drying and grinding.

In the study, highly dispersed wet gas cleaning sludge from the blast furnace of the largest metallurgical enterprise in Russia—Severstal (Cherepovets, Vologda region, Russia)—was used. The native sludge is a black silty sediment (Figure 6a) that forms the bottom deposit of the sludge pond. The pH of the sludge pond supernatant liquid ranged from 7.4 to 8.2, indicating a slightly alkaline reaction of the sludge.

Fifteen subsamples (each ~3 kg) were collected from five different parts of the sludge depository at three depths (0–10, 10–20, and 20–30 cm) to account for vertical and horizontal heterogeneity. Sludge samples were taken from these different parts, averaged (combined into one composite sample with total wet mass ~45 kg), filtered using a vacuum filter, and dried at room temperature for 48 h. The dry sample was homogenized using a Fritsch Pulverisette 2 laboratory mill (Fritsch, Idar-Oberstein, Germany) (Figure 6b). The dry powder was stored in a sealed plastic bag at room temperature. Batch homogeneity was assessed by analyzing three random subsamples of the dried powder using energy-dispersive X-ray spectroscopy (EDS) on the JEOL NeoScope JCM-7000 scanning electron microscope (JEOL, Ltd., Akishima, Tokyo, Japan); the relative standard deviations for Fe and Zn were 2.5% and 1.2%, respectively, confirming good homogeneity.

The microstructure and elemental composition of the sample were studied using a JEOL NeoScope JCM-7000 scanning electron microscope (Japan). X-ray structural studies were performed at room temperature on an X-ray diffractometer (Bruker, AXS, Pforzheim, Germany). The dispersion composition was analyzed using the dynamic light scattering method on a Zetasizer Nano ZS device (Malvern Instruments Ltd., Malvern, UK).

### 4.2. Test Object

The test plant was the sugar beet *Beta vulgaris* L. ssp. vulgaris var. saccharifera Alef., variety “Lgovskaya Odnosemenaya 52”, obtained from the All-Russian Research Institute of Sugar Beet named after A. L. Mazlumov (VNISS, Ramonsky District, Voronezh Region, Russia). Sugar beet is a highly effective model and agent for studying heavy metal contamination due to its strong phytoremediation capabilities, high tolerance to toxins like Cd, Pb, Zn and others, and ability to thrive in polluted soils [74,75].

### 4.3. Greenhouse Experiment

The greenhouse experiments were conducted in accordance with ISO 11269-2:2012 [76]. Artificial soil consisting of a mixture of sand (69%), kaolin clay (20%), neutral peat (10%), and calcium carbonate (about 1%) with the addition of metallurgical sludge in concentrations of 0.01, 0.1, 1, 10, and 100 g kg^−1^. Substrate without the waste material was used as a control. Adding sludge at the maximum concentration increased the pH of the aqueous extract by 0.5–0.8 (from pH 7.0 ± 0.2 to 7.5 ± 0.2). In other cases, adding sludge had no effect. Before sowing the seeds, the substrate was placed in containers with a volume of 7.2 L (length 0.8 m, width 0.3 m, height 0.3 m). Thirty seeds were sown per container. All trials were conducted using 3 independent biological replicates with 3 technical replicates.

The following indicators were recorded: germination capacity, root weight and leaf weight. Cultivation was carried out for 12 weeks. At the end of the experiment, to measure morphometric parameters, 20 random plants were selected from each container (*n* = 60 in one technical replicate). The activity of antioxidant system enzymes (PPO, POD) in plant leaves was analyzed by spectrophotometry using a Multiskan Sky device (Thermo Fisher Scientific, Waltham, MA, USA). To evaluate the enzyme activity, 15 plants were randomly selected from each box and divided into 3 samples of 5 plants each. PPO activity was determined using a spectrophotometric method based on measuring the optical density of reaction products formed during the oxidation of pyrocatechol over a specified period of time. For this purpose, plant material was homogenized in phosphate buffer (pH 7.4) with the addition of polyamide, and the mixture was centrifuged. The supernatant was mixed in a cuvette with catechol solution and phosphate buffer, and the change in optical density was recorded at a wavelength of 420 nm. PPO activity was calculated in activity units per gram of dry matter. POD activity was determined using a method based on measuring the oxidation rate of benzidine by hydrogen peroxide in the presence of POD. Plant material was homogenized in acetate buffer (pH 5.4). The resulting homogenate was centrifuged, and the kinetics of benzidine oxidation by hydrogen peroxide in the presence of POD contained in the supernatant were studied. POD activity was determined by the increase in optical density of the benzidine oxidation product, measured at a wavelength of 590 nm. Enzyme activity was also calculated in activity units per gram of dry matter [77,78]. Thus, 3 measurements were carried out per container (i.e., in one technical repetition *n* = 9).

### 4.4. Field Experiment

#### 4.4.1. Experimental Conditions

Field studies were conducted at Michurinsky State Agrarian University (Michurinsk, Tambov region, Russia). The experiment period was characterized by dry, hot weather with minimal precipitation and an average air temperature of 25 °C. The soil of the agrobiological station is alluvial-meadow, saturated, gley, medium loamy. Agrochemical properties of soil from experimental plots are shown in Table 4.

Prior to sowing, the site was leveled. Soil cultivation included spring plowing to a depth of 20 cm and cultivation. The experimental plot was divided into rectangles with an area of 10 m^2^ each, to which different doses of sludge were applied, and buffer zones of 1 m were left between adjacent plots to prevent cross-contamination. To control for soil heterogeneity and environmental conditions, the experiment was conducted using a randomized complete block design. The experiment was repeated three times.

Sludge was applied before sowing seeds. Sludge doses of 0.5 t ha^−1^, 2 t ha^−1^, and 4 t ha^−1^ were used for the study. The application rates were selected based on the greenhouse experiment results, with the highest dose (4 t ha^−1^) specifically chosen to represent a potential worst-case scenario for a single application, allowing us to assess the margin of safety regarding heavy metal accumulation. The sludge was applied by uniform dry scattering followed by cultivation (plowing depth 25–30 cm). Given the high NPK content of the soil, no additional fertilizers were applied. Plant protection products were not used. Baseline soil metal concentrations are presented in Table 3 for the control plots, as these plots received no sludge and were sampled immediately before the experiment.

The seeding rate was approximately 120 seeds per 10 m^2^, with a seeding depth of 3–4 cm.

#### 4.4.2. Performance Indicators

Intermediate accounting indicators: dimensional indicators (plant height, leaf area), net photosynthetic productivity (dry plant mass increase in grams per unit time (day) per unit leaf area (m^2^). For the analysis, three to five, depending on the parameter, random plots of 1 m^2^ were taken from each experimental plot, from which plants were selected. During the analysis, the height of 50 plants from the plot was measured (10 plants from five 1 m^2^ areas, *n* = 50); plant dry weight and leaf area were measured for 30 plants from three 1 m^2^ plots (10 plants per 1 m^2^) from the total plot.

To determine net photosynthetic productivity (NPP), plant samples were taken and their total mass, the mass of individual organs, and leaf area were determined. Next, net assimilation (in g m^−2^ per day) was calculated using Formula (1):

NPP = W_2_ − W_1_/0.5(*L*_1_ + *L*_2_), (1)

where W_1_ and W_2_ are the average dry weight of 10 plants at the beginning and end of the reference period; (W_2_ − W_1_) is the increase in dry weight per day; L_1_ and L_2_ are the average leaf areas of 10 plants at the beginning and end of the period, m^2^; 0.5(L_1_ + L_2_) is the average active area of leaves during the experiment.

At the end of the experiment, the mass of storage roots (10 root crops were weighed from five 1 m^2^ plots, i.e., *n* = 50 in three replicates), yield (root crop mass from 1 m^2^ × five plots from the main plot, i.e., *n* = 5 in three replicates), and soluble solids content (“%Brix”) in storage roots were determined. The soluble solids content was determined according to the principles of ISO 2173:2003 [79] using a PAL-1 portable refractometer (Atago Co., Ltd., Itabashi, Tokyo, Japan), serving as a reliable indicator of sugar accumulation. Although refractometric determination of °brix is an indirect method that measures total soluble solids rather than individual sugars, it has been widely demonstrated to strongly and reliably correlate with polarimetric sucrose content in sugar beet juice, making it a standard, universally accepted proxy for routine agricultural and quality screening [80]. To determine soluble solids content, we used squeezed sugar beet juice placed on a refractometer prism. An averaged juice sample from 10 root crops from a 1 m^2^ plot was analyzed; from the main plot (10 m^2^), three random areas were taken. Measurements were performed three times for each averaged sample, thus yielding 9 measurements per main plot. To obtain the juice, the root crops were thoroughly washed with tap water and cleaned. They were then cut into smaller pieces and homogenized using a blender to a puree consistency. The mass was passed through a vacuum filter, after which the resulting juice was centrifuged at 10,000× *g* for 10 min in a MiniSpin Plus centrifuge (Eppendorf, Hamburg, Germany) to completely remove suspended particles.

#### 4.4.3. Analysis of Heavy Metal Content

An analysis of heavy metal accumulation in leaves, beet roots and soil from the experimental plots was conducted in order to assess the safety of using blast furnace sludge as a source of micronutrients in crop production. The analysis was performed using atomic absorption spectroscopy on an MGA-1000 device (Lumex, Saint Petersburg, Russia).

##### Analysis of Plant Samples

At the end of the experiment, we analyzed the accumulation of hazardous metals (lead, cadmium, and zinc), as well as iron, which is the main component of the sludge (more than 40%). Five spot samples were collected from each plot (10 m^2^) for analysis. Thus, 15 samples (five samples from each of the three plots) were analyzed for heavy metal content in each treatment. The average sample weight was 1 kg. The analysis was performed separately for leaves and root crops. Heavy metals in the plant samples were determined in their ash solutions.

The samples were dried in an oven at 60–65 °C until air-dry. The dry sample was ground in a mill and sieved through a 2 mm sieve. The dry sample for analysis weighed 100 g.

Plant samples were mineralized using dry ashing. For this, 20 g of the test sample was placed in a crucible, placed in a cold oven, and the temperature was raised to 250 °C (until smoke appeared). After the smoke ceased, the oven temperature was raised to 525 ± 25 °C and calcined for 3 h.

Metal content was determined in the ash solution after mineralization. The ash was moistened with a few drops of bidistilled water, then 10 cm^3^ of dilute nitric acid (1:1) was added to the ash using a dispenser. The crucible was covered with a watch glass and heated on an electric hotplate for 30 min, avoiding splashing of the residue. The crucible contents were filtered into a 50 cm^3^ volumetric flask through a “white ribbon” filter (Ecros, Saint Petersburg, Russia), pre-washed with dilute HNO_3_. The crucible and filter were rinsed several times with hot bidistilled water, bringing the solution volume to the mark.

The following analytical wavelengths were used for analysis: zinc—213.8 nm, lead—217.0 nm, cadmium—228.8 nm, and iron—248.3 nm.

All heavy metal and micronutrient concentrations in the plant samples were calculated and expressed strictly on a dry-weight basis.

##### Soil Sample Analysis

Soil samples were collected from the same areas as the plants. Combined soil samples were compiled from individual soil samples collected using the envelope method (four points in the corners of the plot and one in the center). A total of nine samples from each treatment were analyzed (three combined samples from each plot, measuring 10 m^2^ × 3 plots).

The collected soil samples were ground in a large porcelain mortar and sifted through a nylon sieve with a 1 mm mesh diameter. Soil matrix digestion was performed using an MS-10 microwave sample preparation system (Volta, Saint Petersburg, Russia). The samples were also analyzed on an atomic absorption spectrometer using hollow cathode lamps specific for the target metal (Pb, Mn, Cu, Co, Zn, Cr) (Lumex, Saint Petersburg, Russia).

The safety of the sugar beet roots for consumption was assessed by comparing the concentrations of toxic elements (Pb, Cd) with the MPC for food products established by the international sanitary norms and regulations [34,35,36]. Soil contamination was evaluated against the MPC for soils as per the same regulatory document.

##### Quality Assurance and Quality Control (QA/QC)

To ensure the accuracy and reliability of the analytical data, a rigorous quality control procedure was implemented. Method blanks (reagent blanks) were prepared with each batch of samples (one blank per 10 samples) using the same digestion reagents and procedures; no matrix contamination was detected. Multi-element calibration standard solutions (traceable to NIST standards) were used to construct calibration curves. The linearity of the calibration curves was exceptional, with correlation coefficients (R^2^) exceeding 0.999 for all analyzed elements. The analytical accuracy and precision were validated using the Certified Reference Material (CRM) Tomato Leaves (NIST SRM 1573a) [81] (Sigma-Aldrich, St. Louis, MO, USA). The recovery rates for the targeted elements from the CRM were within the acceptable range of 92–105% (specifically, 94% for Pb, 96% for Cd, 98% for Zn, and 102% for Fe). The limits of detection (LOD) and limits of quantification (LOQ) for the atomic absorption method on the MGA-1000 device were calculated based on a signal-to-noise ratio of 3 and 10, respectively, using 10 blank replicates. The specific values were as follows:

Lead (Pb): LOD = 0.01 mg/kg; LOQ = 0.03 mg/kg.

Cadmium (Cd): LOD = 0.002 mg/kg; LOQ = 0.006 mg/kg.

Zinc (Zn): LOD = 0.05 mg/kg; LOQ = 0.15 mg/kg.

Iron (Fe): LOD = 0.10 mg/kg; LOQ = 0.30 mg/kg.

### 4.5. Processing of Results

The descriptive statistical methods used in the work included the calculation of the arithmetic mean (M) and standard deviation (S) in Excel 2007 MS Office 2007 (Microsoft, Redmond, WA, USA). Data distribution was checked for normality using the Shapiro–Wilk test, and homogeneity of variances was assessed with Levene’s test. To compare multiple treatment groups, one-way analysis of variance (ANOVA) was performed, followed by Tukey’s honestly significant difference (HSD) post hoc test for pairwise comparisons. Differences were considered statistically significant at *p* < 0.05. Variants that share the same letter (a, b, c…) are not statistically different, while groups that do not share any letters have statistically significant differences.

## 5. Conclusions

This study demonstrates that highly dispersed blast furnace sludge, a large-tonnage metallurgical waste, has potential for recycling as a micronutrient source for sugar beet under controlled conditions. At application rates of 0.5–2 t ha^−1^, the sludge increased root crop yield (up to 1.5-fold) and sugar content (up to 1.4-fold) compared to untreated controls. Additionally, the sludge exhibited phytoprotective effects, reducing leaf necrosis under arid field conditions and lowering oxidative stress markers (PPO, POD) in greenhouse experiments.

A single application at the tested rates did not result in critical food safety or soil integrity issues within the study period: lead and cadmium concentrations in beetroots and soil remained below international regulatory limits (EU 2023/915, FAO/WHO), and zinc levels in beetroots (≤10 mg kg^−1^) were an order of magnitude below the FAO/WHO guideline. However, soil zinc accumulation represents the primary constraint of this approach, requiring strict long-term monitoring. Preliminary screening-level estimates suggest that a repeated annual application of 2 t ha^−1^ could approach the soil MPC for zinc within 4–5 years, disregarding potential crop removal or leaching.

Blast furnace sludge composition varies between smelters, so the results reported here may not automatically apply to sludge from other sources. Any agricultural use requires prior characterization of metal content, particle size, and regulatory compliance for each new batch.

From a circular economy perspective, repurposing blast furnace sludge is a promising option to valorize a problematic waste stream and reduce dependence on synthetic micronutrients. However, broad agricultural adoption is not yet warranted. To validate safety and efficacy, future research must focus on: (1) long-term field trials across diverse soil types to model zinc accumulation kinetics; (2) validation on other crops (cereals, legumes, vegetables); and (3) development of standardized characterization protocols for sludge batches. Addressing these limitations is essential to determine if recycling blast furnace sludge can serve as a viable resource-efficient pathway in green metallurgy.

## Figures and Tables

**Table 1:** Elemental composition of the sludge sample.

Element	Mass %
C	9.2 ± 1.1
O	18.4 ± 1.1
Na	3.97 ± 0.08
Mg	0.63 ± 0.02
Al	2.53 ± 0.11
Si	6.50 ± 0.26
P	0.14 ± 0.01
S	2.80 ± 0.06
K	0.78 ± 0.06
Ca	4.72 ± 0.22
Ti	0.29 ± 0.03
Fe	41.6 ± 1.6
Zn	8.5 ± 0.7

**Table 2:** Heavy metal content in sugar beet plants (mg kg^−1^ dry weight).

Groups	Lead	Cadmium	Zinc	Iron
Leaves				
Control	0.25 ± 0.01a	0.025 ± 0.002b	3.13 ± 0.2	18.2 ± 0.6
0.5 t ha^−1^	0.19 ± 0.02a	0.027 ± 0.003b	7.06 ± 0.6	52.6 ± 0.4
2 t ha^−1^	0.21 ± 0.03a	0.024 ± 0.008b	5.35 ± 0.9c	37.6 ± 0.5d
4 t ha^−1^	0.29 ± 0.08a	0.029 ± 0.006b	5.34 ± 0.7c	33.3 ± 0.2d
Roots				
Control	0.17 ± 0.03e	0.021 ± 0.002f	6.39 ± 0.4g	31.5 ± 0.2i
0.5 t ha^−1^	0.15 ± 0.02e	0.031 ± 0.006f	5.49 ± 0.8g	32.3 ± 0.1i
2 t ha^−1^	0.21 ± 0.04e	0.029 ± 0.004f	9.1 ± 0.6h	31.6 ± 0.3i
4 t ha^−1^	0.18 ± 0.01e	0.025 ± 0.002f	8.04 ± 0.2h	29.8 ± 0.3i
MPC ^1,2^				
	0.5	0.06	10	-

^1^ TP TC 021/2011 Technical Regulations of the Customs Union “On food safety” [35]. ^2^ Regulation (EU) 2023/915 on maximum levels for certain contaminants in food [36]. Values followed by the same letter are not significantly different.

**Table 3:** Heavy metal content in soil (mg kg^−1^) after harvesting crops grown using highly dispersed blast furnace sludge.

Groups	Lead	Manganese	Copper	Cobalt	Zinc	Chromium (III)
Control	0.4 ± 0.02a	6.5 ± 0.3b	0.1 ± 0.001c	0.1 ± 0.02d	3.6 ± 0.2	2.49 ± 0.3e
0.5 t ha^−1^	0.31 ± 0.01a	7.7 ± 0.4b	0.11 ± 0.02c	0.14 ± 0.02d	5.07 ± 0.4	3.01 ± 0.2e
2 t ha^−1^	0.39 ± 0.02a	6.5 ± 0.2b	0.17 ± 0.02c	0.16 ± 0.03d	8.2 ± 0.4	3.04 ± 0.5e
4 t ha^−1^	0.43 ± 0.02a	7.8 ± 0.5b	0.44 ± 0.08	0.36 ± 0.03	10.56 ± 0.6	3.08 ± 0.3e
MPC ^1^	6.0	140.0	3.0	5.0	23.0	6.0

^1^ SanPiN (Sanitary Norms and Regulations) 1.2.3685-21 [37]. Values followed by the same letter are not significantly different.

**Table 4:** Agrochemical parameters of soil.

Depth, cm	Macronutrient Content, mg/100 g			C_org_, %	pH_aw_	pH_as_	H	S	V, %
	P_2_O_5_	K_2_O	N				μ/100 g		
0–10	22.4	35.4	26.3	4.2	6.8	5.9	1.3	48.5	97.4
10–17	17.4	28.3	21.8	3.9	6.9	5.6	0.9	44.3	98.0
17–30	11.2	24.4	22.1	1.4	7.0	5.8	0.7	43.3	98.4

N—alkaline hydrolyzable nitrogen, C—organic carbon, pH_aw_—acidity of water extract from the soil (actual acidity), pH_as_—acidity of saline (KCl) extract from the soil (exchangeable acidity), H—hydrolytic acidity, S—exchangeable bases, V—the degree of saturation of the soil-absorbing complex with bases.

## Data Availability

The original contributions presented in this study are included in the article. Further inquiries can be directed to the corresponding author.
